# Effectiveness of growth promoters for the seagrass (*Cymodocea nodosa*) restoration

**DOI:** 10.3389/fpls.2025.1507804

**Published:** 2025-06-03

**Authors:** Giuliana Marletta, Domenico Sacco, Roberto Danovaro, Silvia Bianchelli

**Affiliations:** ^1^ Dipartimento di Scienze della Vita e dell’Ambiente, Università Politecnica delle Marche, Ancona, Italy; ^2^ National Biodiversity Future Centre, Palermo, Italy

**Keywords:** habitat-forming species, seagrass, Mediterranean Sea, complementary actions, active restoration

## Abstract

Seagrass meadows are regressing due to the cumulative impacts that affect coastal ecosystems worldwide. Seagrass restoration has been repeatedly proposed to reverse this trend, although with contrasting results due to the difficulty in maintaining the transplanted rhizomes. Enhancing the vegetative propagation of the rhizome plantings (e.g., employing growth-promoters) could represent a reliable tool to increase the success of seagrass restoration. Here we tested the effects of physio-activators, as plant growth-promoting bacteria (PGPB), and synthetic hormones, as plant growth regulators (PGRs), on a seagrass species to assess their potential utilization to enhance restoration efficiency. We conducted two separate experiments in aquaria on *Cymodocea nodosa* fragments: in the first one, the fragments were exposed to PGRs for six weeks, while in the second experiment, the fragments were exposed to PGPB for four weeks. For each experiment (PGRs and PGPB), the formation of new roots and new leaves, the survivorship, and the trend of maximum leaf length were compared between the treated and control (not exposed to PGRs or PGPB) fragments. It was observed that only the PGPB had a significant effect on the fragments’ survivorship (90% in treated fragments vs. 25% in control ones) and contributed significantly to the formation of new leaves and roots of *C. nodosa* fragments. On the contrary, in the experiments with PGRs, no significant effects were observed between treated and control fragments, and both showed a survivorship of 100% at the end of the experiment. Our study showed that the application of growth-promoters (particularly PGPB) on fragments could increase their survival and the formation of new roots and leaves. Therefore, the use of PGPB on *C. nodosa* fragments can allow their re-employment in restoration interventions, without damaging the individuals of natural populations.

## 1 Introduction

Seagrasses are marine angiosperms playing a crucial role in temperate and tropical coastal habitats, as they provide important ecosystem services (Costanza et al., 1997; Boudouresque et al., 2021). They include 67 species worldwide, 7 of which are present in the Mediterranean Sea: *Posidonia oceanica* is the only endemic species of the Mediterranean Sea, whereas *Cymodocea nodosa*, *Zostera marina* and *Z. noltei* show a broader distribution at temperate latitudes (Boudouresque et al., 2009; Ruiz et al., 2009), *Ruppia maritima* is almost entirely restricted to brackish lagoons and salt marshes (Shili et al., 2007), and *Halophila stipulacea* and *H. decipiens* are non-indigenous species (Winters et al., 2020; Gerakaris et al., 2020).

Over the last decades, seagrasses have severely declined due to anthropogenic activities, and only a few meadows recovered (Waycott et al., 2009; Orth et al., 2006; de los Santos et al., 2019; Sinclair et al., 2021). As a result of direct and indirect anthropogenic pressures, seagrass meadows are shrinking their global distribution at a rate ranging from 1% per year (till 1940) to 7% per year (after 1990, Waycott et al., 2009). In the Mediterranean Sea, the main causes of this decline are coastal development, increased maritime traffic, eutrophication, and chemical contamination (Pergent et al., 2014). Between 1973 and 1989, it was observed that the seagrass meadows of the northern Adriatic Sea were subjected to a decrease in extension due to the explosion of coastal urbanization, the intensification of tourism flow, and a significant increase in eutrophication caused by the Po River flow (Danovaro et al., 2020). Even a long-term analysis conducted from 1869 to 2016 in the European Seas showed that between the 1970s and 1980s, there was a sharp decline in seagrass meadows due to disease, deteriorated water quality, and coastal development (de los Santos et al., 2019).

Climate change and extreme events such as heat waves can exacerbate the rapid loss of shoot density and increase the energy needed to reproduce and produce defense compounds (Pergent et al., 2008). In the Greek Seas, the increase in the thermal regime over two decades (1997-2018) was followed by a decline in *P. ocenica* production (Litsi-Mizan et al., 2023). Stipcich et al. (2022) tested the effects of current and future Marine Heatwaves (MHWs) through a manipulative experiment in Sardinia (Italy) and observed significant changes in the morphological and biochemical variables of *P. oceanica* shoots. They found that current and future MHWs could have similar effects, with a difference depending on the intensity of the waves: the number of leaves, the maximum leaf length, and lipid content decreased, while the leaf necrosis and carbohydrate content increased (Stipcich et al., 2022). Beca-Carretero et al. (2024) applied a novel ecological and spatial model, considering two climate scenarios (RCP 2.6 and RCP 8.5) projected from 2020 to 2100 in four different regions within the Mediterranean (West, Central West, Central East, East Mediterranean). They foresee that with rising temperature and salinity, the habitat of *P. ocenica* will be lost and colonized by more resilient species such as *C. nodosa* and the invasive species *H. stipulacea*. Under the worst scenario (RCP 8.5), the most negative effects have been foreseen in warmer regions (Central and East Mediterranean), while the western region will represent a refuge area for *P. oceanica* (Beca-Carretero et al., 2024).

In recent decades, thanks to the enforcement of conservation measures (e.g., Habitat Directive, Water and Marine Strategy Framework Directives, Marine Protected Areas institution), some meadows displayed encouraging signs of stabilization or recovery (de los Santos et al., 2019). Moreover, in the last two decades, several restoration interventions have been implemented (Orth et al., 2006; Marbà et al., 2014). Although active restoration is considered an increasingly reliable approach to enhancing the recovery of seagrass ecosystems, to date, restoration results have not always been fully successful, due to many factors, such as the seagrass’s low growth rate and complex reproduction cycle (Bekkby et al., 2020), site selection (Paling et al., 2009; Bayraktarov et al., 2016), and used methodology (Da Ros et al., 2020).

Previous studies demonstrated that one of the major causes of restoration failure is the difficulty in maintaining *in situ* the transplanted rhizomes (Lepoint et al., 2002). During vegetative propagation, the formation of adventitious roots enables the plant to remain firmly attached to the substrate and to absorb the nutrients (Duguma, 1988; Swamy et al., 2002). Therefore, techniques enabling the development of a robust root system could facilitate the vegetative expansion of the transplanted rhizomes (Balestri and Lardicci, 2006). To accelerate vegetation expansion and improve transplant success (Loquès et al., 1990; Balestri et al., 1998; Balestri and Cinelli, 2001), other studies proposed the transplant of entire plants with the surrounding sediments contained in organic and biodegradable containers (Da Ros et al., 2020).

Several studies have shown the role of physio-activators, such as plant growth-promoting bacteria (PGPB), and synthetic hormones, such as plant growth regulators (PGRs), in the increase of the growth, development, and germination abilities over a wide range of terrestrial plants (Russo and Berlyn, 1990; Crunkilton et al., 1994; Swaminathaan and Srinivasan, 1996; Ortíz-Castro et al., 2009; Small and Degenhardt, 2018; Kumari et al., 2023). These molecules promote vegetative propagation, enhancing root and leaf formation and growth (Salisbury and Ross, 1992; Crunkilton et al., 1994; Katiresan and Moorthy, 1994; Munoz, 1995; Swaminathaan and Srinivasan, 1996). Moreover, the PGPB increase plant resilience against abiotic stressors (i.e., salinity, drought) and protect plants from diseases, inducing defense systems (Adhikari et al., 2001; Bloemberg and Lugtenberg, 2001; Weyens et al., 2009; O’Callaghan, 2016; Sánchez-López et al., 2018; Rossi et al., 2021).

Only a few studies investigated the effects of PGRs on Mediterranean seagrasses, with promising results on the plants’ growth (Munoz, 1995; Balestri and Bertini, 2003; Balestri and Lardicci, 2006; Balestri et al., 2011), but the effects of PGRs and PGPBs have never been investigated for enhancing the restoration efficacy on Mediterranean seagrasses (Loquès et al., 1990; Munoz, 1995), particularly for those interventions requiring *ex-situ* maintenance or growth and reproduction. The use of plant promoters could indeed increase the restoration effectiveness (Cebrian et al., 2021; Smith et al., 2023). The present study aims to test the effects of PGRs and PGPB on the survival and growth of *C. nodosa*. To avoid any impact on natural populations we explored their potential to produce new shoots and roots from fragmented plants that could represent a potentially important source of plants for restoration interventions (Campbell, 2002).

## 2 Materials and equipment

### 2.1 The species

*C. nodosa* is a pioneer seagrass (Marín-Guirao et al., 2016), forming dense meadows in shallow waters across the Mediterranean Sea and the Northeast Atlantic, including the Canary Islands (Reyes et al., 1995; Pavón-Salas et al., 2000; Alberto et al., 2008; Cunha and Araújo, 2009). This dioecious species is characterized by horizontal rhizomes, which at each node bring a short vertical rhizome ending dorsally with a leaf tuft of 3–4 leaves, and ventrally with irregularly branched roots. The leaves have a ribbon shape and feature 7–9 parallel ribs, and a rounded and obtuse apex (Rodríguez-Prieto et al., 2013). For its role in ecosystem structuring, *C. nodosa* is considered the second most important seagrass species in the Mediterranean Sea, after *P. ocenica*. It shows a wide environmental tolerance: along the sandy coasts, it grows in shallow and sheltered areas, in clear waters, also beyond the deep limit of *P. oceanica*, but also on dead *matte* of *P. oceanica* (Rodríguez-Prieto et al., 2013).

### 2.2 Samples’ collection

**Figure 1:**
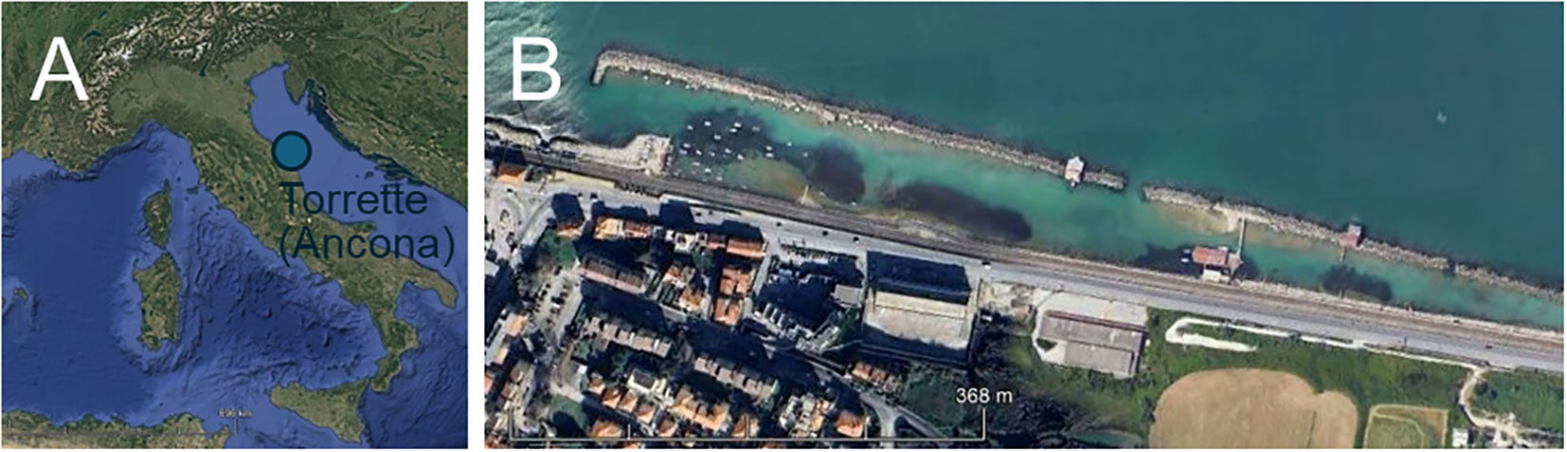
**(A)** Location of the study area in the Mediterranean Sea; **(B)** Detail of the study area (43°36’36”N, 13°27’30”E). Map created using the Free and Open Source QGIS.

During October and November 2023, two samplings were conducted at Torrette site (near Ancona city, North-western Adriatic Sea; 43°36’36”N, 13°27’30”E; **Figures 1A, B**). In this site, between the coastline and the breakwaters, there is a rock pool formed by artificial reefs, hosting a meadow of *C. nodosa* (ca. 1 hectare wide) at about 0.5 m depth on muddy substrate.

During the two samplings, a total of 15 and 40 fragments were collected, respectively, stranded, or manually collected by hand from plants. Since *C. nodosa* is under conservationist attention, the minimum number of fragments has been collected to run the experiments. For each sampling, the fragments were transported (in transportable aerated tanks, with seawater collected *in situ*) to the Aquarium Facility of the Department of Life and Environmental Science (Polytechnic University of Marche, Ancona; 43°58’N; 13°51’E), located 8 km from the sampling site, for ca. 14 minutes of transport.

Once arrived, the fragments were acclimatized for one hour through a slow mixing between the seawater used for the transport and the water of the tanks (previously prepared at the same temperature and salinity). The fragments collected during the first and second sampling were used for testing the effect of PGRs and PGPB, respectively, through 2 experiments in mesocosms, running separately.

## 3 Methods

### 3.1 Maintenance in the mesocosms

For each experiment, 2 separate aquaria systems were used, each consisting of 2 tanks (volume 50 L, each): one aquarium for the treatment (2 tanks with fragments exposed to PGRs or PGPB, in the first and second experiment, respectively) and 2 used as control (2 tanks with fragments).

The LSS (Life Support System) was used to maintain the plants in the mesocosms. It consists of aquaria, a reserve in which there are three socks of 100 μm for mechanical filtration, and immersed razor clams for biological filtration. Fluorescent lamps produced 260-nm (λ) UV-C rays, sterilizing the water, damaging nucleic acids, and preventing microbes’ proliferation. A Teco TK 500 cooler was used to maintain the temperature. The light intensity was generated by two light-emitting diode lamps (SilverMoon Marine 10,000 K and SilverMoon Universal 6,500 K) 40 cm above the water surface. Irradiance was measured with a Photometer of the Apogee Model MQ-500. The system ensures the maintenance of constant ambient water conditions. The photoperiod was set to a 12:12 h light: dark cycle, with an intensity of 80-100 µmol photons m^−2^ s^−1^ to simulate the environmental conditions present during sampling. Temperature, salinity, pH, and light intensity were measured at the sampling site and were set up in aquaria following Marín-Guirao et al. (2011) for the same sampling period. These parameters were maintained throughout the experiments: temperature was 20 ± 1°C, salinity was 37, and pH was 8.2. Furthermore, for routine system maintenance, water loading and unloading, lights, movement pumps, a cooler, and any water leaks at the pipe joints were checked. To ensure the sterilization of the system, the socks were washed, tubs were siphoned to remove organic debris, and 20% of the seawater was exchanged every week. The replacement water was prepared using artificial salts.

### 3.2 Experimental design

**Figure 2:**
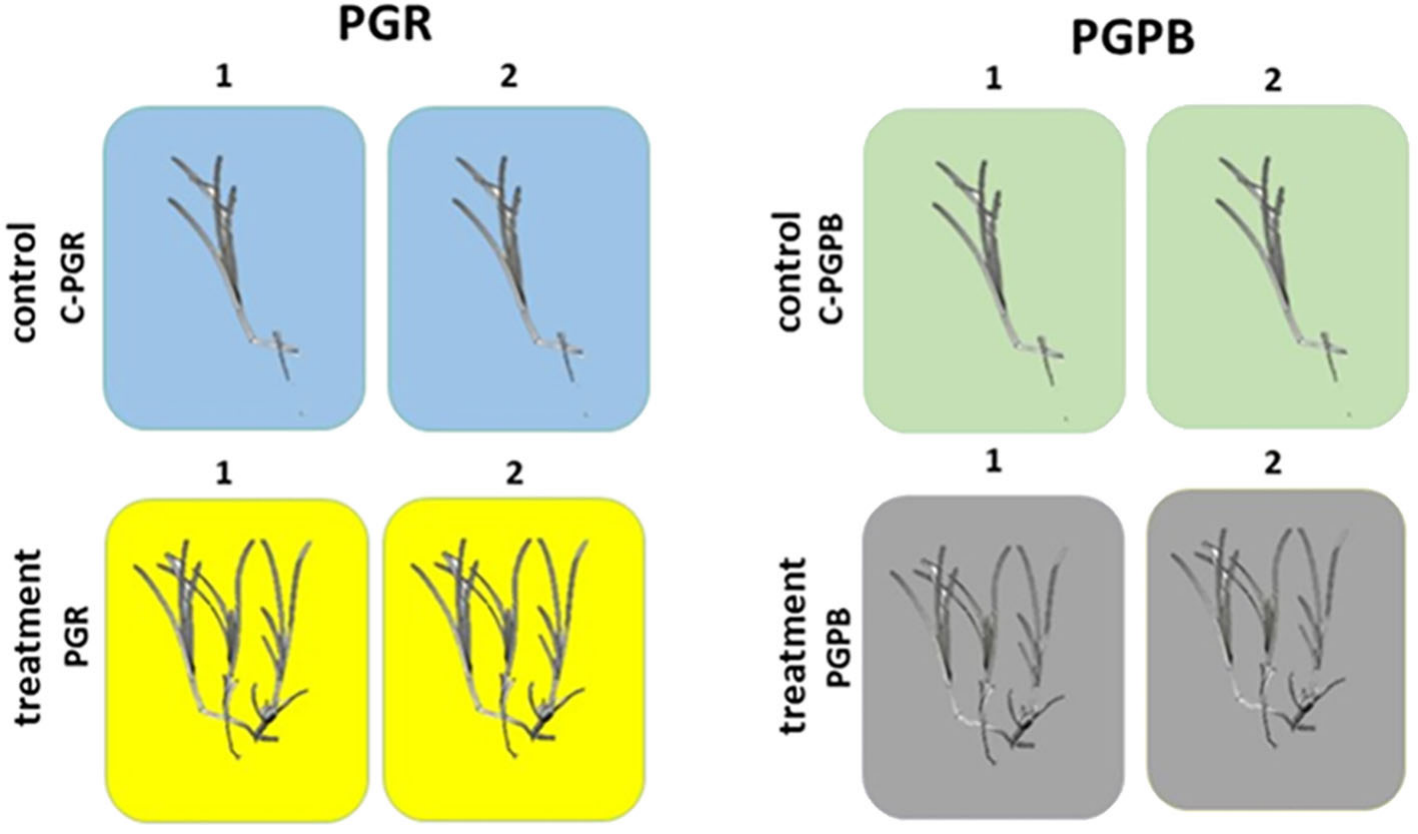
Experimental design for the PGRs and PGPB experiments. In each experiment, *C. nodosa* fragments were exposed to PGR (left panel) or PGPB (right panel) (n = 2) and compared to fragments not exposed (n = 2). Treatment = treated fragments (with PGR or PGPB, depending on the experiment); Control = fragment not exposed during each experiment (C-PGR and C-PGPB, respectively); 1 and 2 = code of the tank used.

The first experiment consisted of 4 tanks (volume 50 L, each), 2 used for the treatment with PGRs (n = 2) and 2 as control (n = 2). The tanks used as control were labelled as C-PGRs1 and C-PGRs2, containing 2 fragments each. Those used for the treatment with PGRs were labelled as PGRs1 and PGRs2, containing 6 and 5 fragments, respectively (**Figure 2**). All fragments were fixed to plastic nets with a small weight to maintain them on the bottom of the tanks. In the tanks PGRs1 and PGRs2 it was added one pill of Gibaifar 10 TB, containing gibberellic acid (GA3), and 80 ml of Sprintex New^®^ L, containing alpha-naphtaleneacetic acid (NAA).

The second experiment consisted of 4 tanks (volume 50 L, each), 2 for the treatment with PGPB (n =2) and 2 as control (n = 2). In this case, tanks used as control were labelled as C-PGPB1 and C-PGPB2. Those used for treatment with PGPB were labelled as PGPB1 and PGPB2 (**Figure 2**). Each tank contained 10 fragments. In this case, all the fragments were fixed to jute nets with a small weight to maintain them on the bottom of the tanks. In the mesocosms PGPB1 and PGPB2, 100 ml of Microtech Triple eco was added, which contains growth-promoting bacteria and cyanobacteria.

In both experiments, all fragments were tagged, photographed, and their growth measured. The following variables were checked once a week, for 6 and 4 weeks for the first and second experiment, respectively: number of shoots, roots, and leaves, and maximum leaf length. The collection of the abovementioned data allowed us to estimate: the number of new roots, new leaves, survivorship (number of individuals showing new leaves or roots), and trend of the maximum leaf length (following Balestri and Lardicci, 2006; Balestri et al., 2011). All the variables were measured for all the individuals in each tank and reported as tanks’ mean ± standard error.

### 3.3 Statistical analyses

To test differences in the abovementioned variables (maximum leaf length, number of new roots and leaves, and survivorship), separately for the 2 experiments, one- or two-way permutational analysis of variance (PERMANOVA) was performed, applying two experimental designs.

For the maximum leaf length, two factors of variance were considered: “time” (fixed, 2 levels: beginning and end of the experiments, corresponding to 6 and 4 weeks for the PGRs and PGPB experiment, respectively) and “treatment” (fixed, 2 levels: control and treatment, with the factor “tank” nested in “treatment”).

All other variables (i.e., number of new roots and leaves, and survivorship) were considered as a single factor of variance in the “treatment” (fixed, 2 levels: control and treated, with the factor “tank” nested in “treatment”) since at the beginning there were no new roots and leaves.

Before PERMANOVA, PERMDISP tests were carried out to test the dispersion among groups: “time x treatment(tank)” for max leaf length or “treatment(tank)” for new roots, new leaves, and survivorship. When PERMDISP was significant, the data were fourth root transformed before PERMANOVA.

Statistical analyses were performed by using the software package PRIMER7 (Clarke and Gorley, 2015).

## 4 Results

All the data are reported in **Table 1**.

**Table 1:** Variables and data measured during the experiments.

			max leaf legth (cm)		n. new roots/ind		n. new leaves/ind		survivorship (n.ind with new roots/leaves)	survivorship (n. new roots or leaves/ind)	
			avg	se	avg	se	avg	se	%	avg	se
PGRs	Ctrl	beginning	34.9	3.1	na		na		na	na	
		after 6 weeks	11.8	1.0	0.4	0.1	1.0	0.8	100	1.4	0.9
	Treat	beginning	32.1	1.7	na		na		na	na	
		after 6 weeks	12.9	4.2	0.5	0.3	0.4	0.2	100	0.9	0.5
PGPB	Ctl	beginning	20.6	5.0	na		na		na	na	
		after 4 weeks	17.3	4.9	0.0	0.0	0.02	0.01	25.0	0.04	0.01
	Treat	beginning	16.3	2.7	na		na		na	na	
		after 4 weeks	10.4	1.9	0.1	0.0	0.1	0.0	90.0	0.2	0.1

Data are reported as mean ± standard error, calculated based on the means values for each tank.

The results of the PERMDISP testing for dispersion among groups for PGRs and PGPB experiments are reported in **Tables 2A, B**, respectively. The results of the PERMANOVA analyses testing for the effect of treatment and time or only treatment, depending on the variable, on all the considered response variables, for PGRs and PGPB experiments, are reported in **Tables 3A, B**, respectively.

**Table 2:** Output of the PERDISP conducted after PGRs (A) and PGPB (B) experiments, on all variables, testing for the dispersion among groups: “treatment(tank) x time” for max leaf length or “treatment(tank)” for new roots, new leaves, and survivorship.

		Max leaf length	New roots	New leaves	Survivorship
A)	PGRs	F: 2,4834 df1: 7 df2: 22	F: 1,4753 df1: 3 df2: 11	F: 1,7814 df1: 3 df2: 11	F: 4,8668 df1: 3 df2: 11
		P: 0.071	P: 0.253	P: 0.267	P: 0,084*
B)	PGPB	F: 1,8131 df1: 7 df2: 72	F: 1,5004 df1: 3 df2: 36	F: 0,96122 df1: 3 df2: 36	F: 0,23953 df1: 3 df2: 36
		P: 0.083	P: 0.222*	P: 0.427*	P: 0.863*

*data fourth root transformed. df, degree of freedom; F, F statistic; P, p value.

**Table 3:** Output of PERMANOVA conducted after PGRs (A) and PGPB (B) experiments, testing for differences between treatment (tanks) and times (for the max leaf length) or treatment (tanks) (for new root, new leaves, and survivorship).

			Source	df	MS	F	P
A)	PGRs	Max leaf length	Time	1	2617.20	23.20	<0.05
			Treatment	1	4.00	0.07	ns
			Tank (Treatment)	2	33.90	0.22	ns
			Time x Treatment	1	22.95	0.20	ns
			Time x Tank (Treatment)	2	102.21	0.67	ns
			Residual	22.0	151.55		
		New roots	Treatment	1	9.84	1.09	ns
			Tank (Treatment)	2	11.07	7.03	<0.05
			Residual	11	1.58		
		New leaves*	Treatment	1	0.01	0.02	ns
			Tank (Treatment)	2	0.42	1.48	ns
			Residual	11	0.28		
		Survivorship*	Treatment	1	0.10	0.36	ns
			Tank (Treatment)	2	0.35	23.70	<0.01
			Residual	11	0.01		
B)	PGPB	Max leaf length	Time	1	146.07	305.83	<0.01
			Treatment	1	504.51	5.15	ns
			Tank (Treatment)	2	97.95	1.21	ns
			Time x Treatment	1	51.04	106.86	<0.05
			Time x Tank (Treatment)	2	0.48	0.01	ns
			Residual	72	80.96		
		New roots*	Treatment	1	1.25	16.27	<0.05
			Tank (Treatment)	2	0.08	0.29	ns
			Residual	36	0.27		
		New leaves*	Treatment	1	3.88	14.85	<0.05
			Tank (Treatment)	2	0.26	1.29	ns
			Residual	36	0.20		
		Survivorship*	Treatment	1	6.07	65.61	<0.05
			Tank (Treatment)	2	0.09	0.44	ns
			Residual	36	0.21		

*data fourth root transformed. Source, source of variance; df, degree of freedom; MS, means of squares; F, F statistic; P, p value.

### 4.1 PGRs treatment

**Figure 3:**
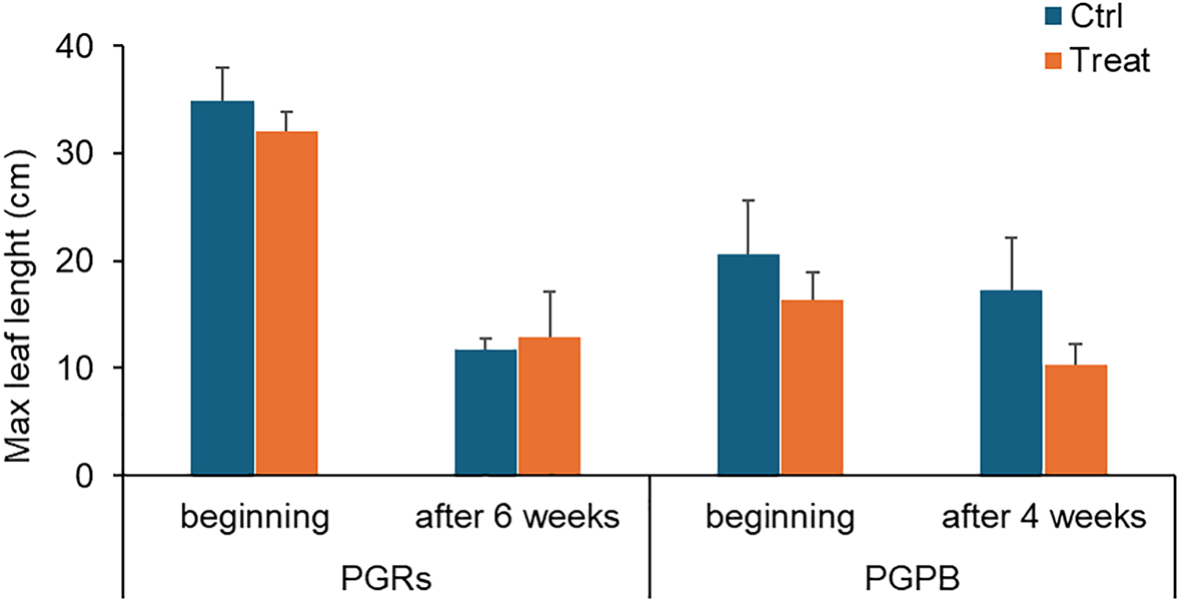
Max leaf length measured in fragments used as control (in light blue) and those exposed to PGRs or PGPB (in orange), at the beginning and after 6 and 4 weeks, respectively. Data (in cm) are reported as the average of values measured in the tanks ± standard error. Ctrl, control; Treat, treatment.

*Maximum leaf length* - A significant effect of time was observed on the maximum leaf length (**Table 2A**). The maximum leaf length was significantly lower at the end of the experiment only in control fragments. However, the values were similar in the controls and treated fragments in the PGRs experiment, both at the beginning and the end of the experiment (i.e., after 6 weeks, **Figure 3**).

**Figure 4:**
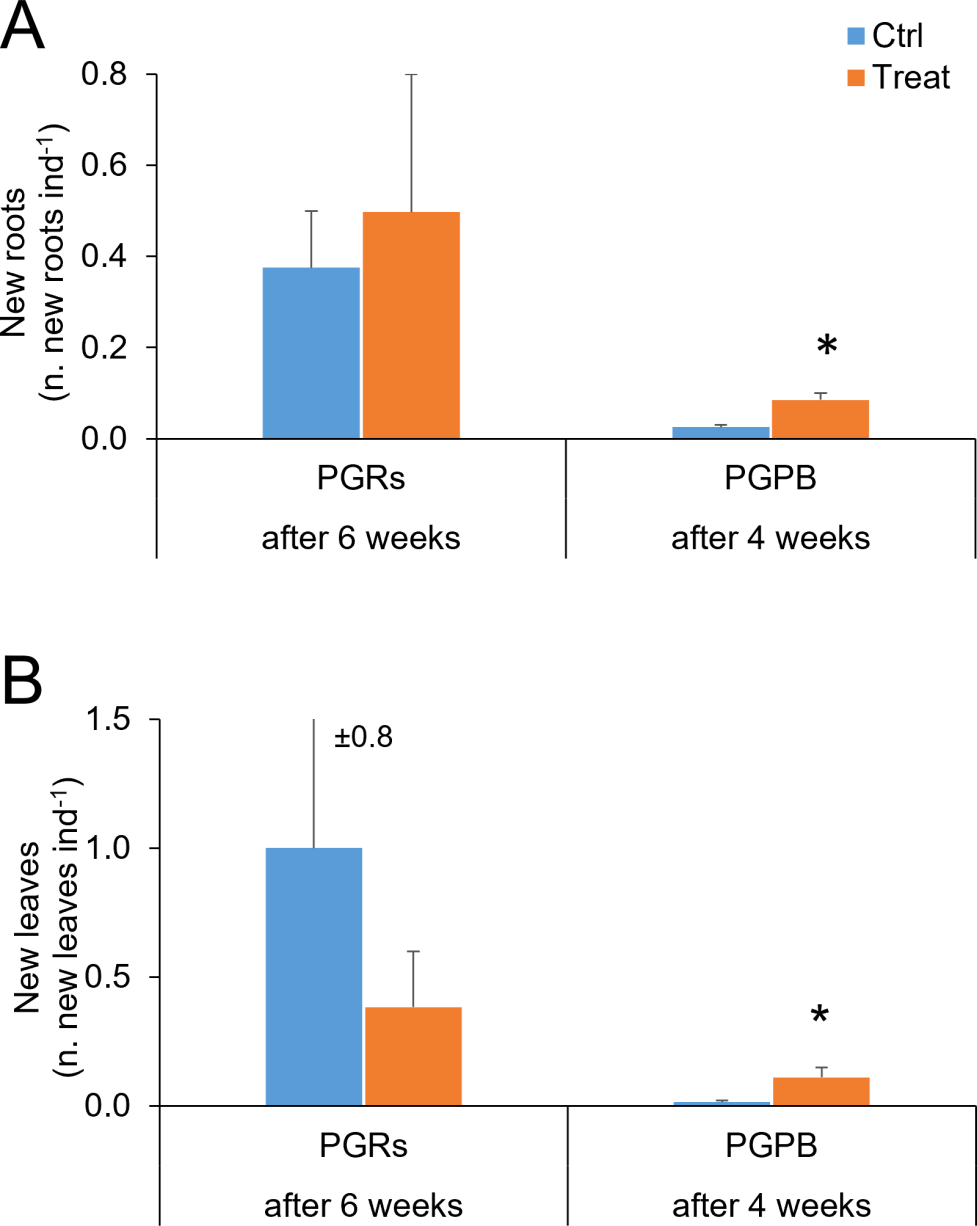
New roots **(A)** and new leaves **(B)** observed in fragments used as control (in light blue) and those exposed to PGRs or PGPB (in orange), after 6 and 4 weeks, respectively. Data (as number of new roots and leaves per fragment) are reported as the average of values measured in the tanks ± standard error. Ctrl, control; Treat, treatment; ind., individual (= fragment); *P < 0.05.

*Formation of new roots and new leaves*– The formation of new roots and new leaves was observed at the end of the experiment (after 6 weeks), both in control and treated fragments. However, no significant differences were observed between control and treated fragments, for both variables (**Table 2A**; **Figures 4A, B**). A significant effect of the factor tank was observed for new roots’ formation.

**Figure 5:**
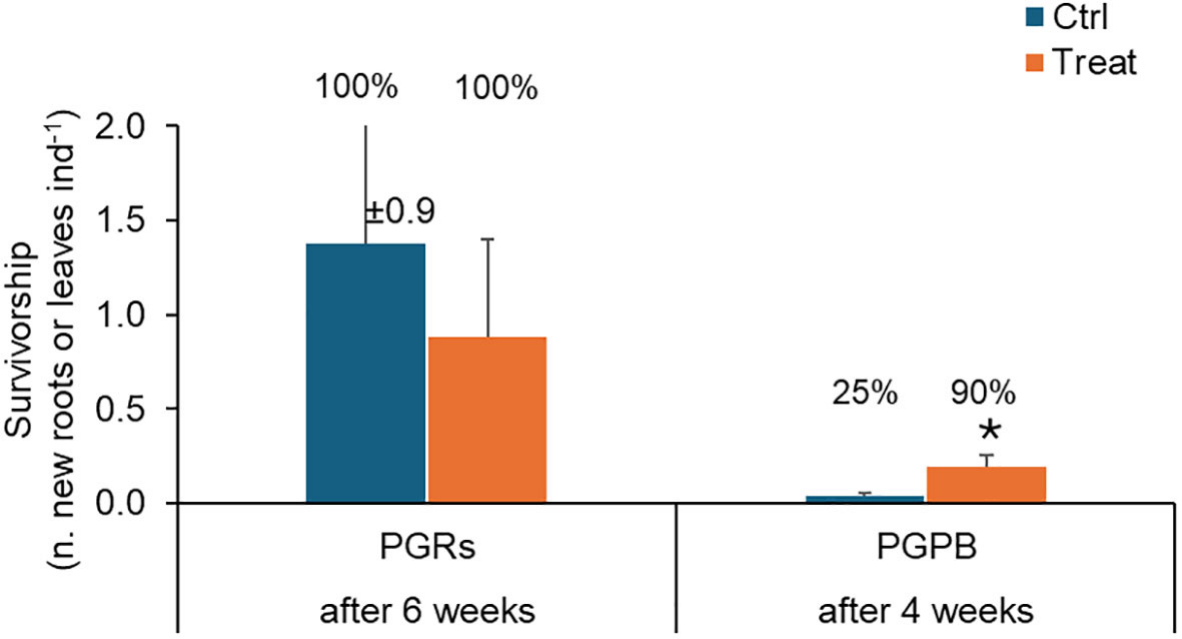
Survivorship observed in fragments used as control (in light blue) and those exposed to PGRs or PGPB (in orange), after 6 and 4 weeks, respectively. Data (as the number of new roots or leaves per fragment) are reported as the average of values measured in the tanks ± standard error. Reported are also the % of individuals developing new roots or leaves. Ctrl, control; Treat, treatment; ind., individual (= fragment); *P < 0.05.

*Survivorship* – At the end of the experiment, in control and treated experimental units, the 100% of individuals showed survivorship (measured as new leaves or roots). However, no significant difference was observed comparing control and treated fragments (**Table 2A**; **Figure 5**). A significant effect of the factor “tank” was observed.

### 4.2 PGPB treatment

*Maximum leaf length* – A significant effect of time and time x treatment was observed on the maximum leaf length (**Table 2B**; **Figure 3**). No significant differences were observed between the control and treated fragments.

*Formation of new roots and new leaves* – The formation of new roots and new leaves was observed at the end of the experiment (after 4 weeks). A significant difference was observed between control and treated fragments, with higher values observed in treated fragments, for both variables (**Table 2B**; **Figures 4A, B**).

*Survivorship* – Overall, in control and treatment experimental units, 25 and 90% of individuals showed survivorship (measured as new leaves or roots). A significant effect of the treatment on survivorship was observed (**Table 2B**), with higher values observed in treated fragments (**Figure 5**).

## 5 Discussion

Mediterranean seagrasses are crucially important species, most of which are protected by international conventions such as the Bern Convention, the SPAMI-Barcelona Convention, and the Action Plan for the Conservation of Marine Vegetation in the Mediterranean Sea. They are also part of the Habitat Directive (Curiel et al., 2021). Due to their role in blue carbon sequestration and the coastal ecosystem functioning, marine meadows’ restoration has been proposed as a tool for climate change mitigation (Orth et al., 2006; Marbà et al., 2014; United Nations Environment Agency, 2019; United Nations Environment Programme, 2019). To upscale these interventions, recent studies highlighted the importance of improving transplanting operations by reducing the cost and increasing intervention efficacy (Boudouresque et al., 2021). Moreover, the restoration intervention should be planned to avoid any possible damage to healthy populations. This can be done only by optimizing protocols, also considering phases implemented *ex situ*, and using laboratory facilities to improve the reproduction or cultivation of individuals used for the outplants or transplants at sea.

Compared to other Mediterranean species, *C. nodosa* offers the greatest chance for restoration success, due to its high tolerance to varying environmental conditions (Bellato et al., 1994; Rismondo et al., 1997; Sfriso and Ghetti, 1998). The formation of new roots allows the vegetative expansion of the plant, increasing the probability of permanent establishment of the new seagrass beds (Balestri and Lardicci, 2006). However, extreme environmental events such as storm surges, which have increased in intensity and frequency due to climate change, can cause the loss of large portions of natural seagrass meadows (Oprandi et al., 2020).

The implementation of growth-promoters has proven to be successful in stimulating the rooting capacity of seagrasses (Balestri and Bertini, 2003; Balestri and Lardicci, 2006). Our study showed that PGPB have a significantly positive effect on *C. nodosa* and its stranded fragments. This response allows more efficient use of stranded fragments of seagrasses for habitat restoration, a strategy successfully used for macroalgal forest restoration (Marletta et al., 2024). This would allow to use only some parts of the plants, avoiding using entire portion of the meadow for the restoration interventions.

The effects of these growth-promoters can depend on the molecule (PGPB or PGRs). Our results indicated survivorship (i.e., formation of new roots or leaves) both in control and treated fragments, suggesting that the cultivation conditions were optimal in the aquaria facility. However, only the PGPB growth-promoters had positive effects on the fragments’ survivorship and contributed significantly to the increase in the formation of new leaves and roots in *C. nodosa*, when compared with control fragments. Therefore, the future use of growth promoters needs to be previously tested, since different promoters (i.e., different molecules) could have a different (or null) effect on the plants’ growth. This is particularly important when planning a cost-effective restoration process, which includes an *ex-situ* phase. In the experiment with PGPB, the fragments had a higher survivorship when exposed to the growth-promoters (90%) than in the control ones (25%). This could be related to the ability of the plant to incorporate the PGPB through the leaves, with a process possibly catalyzed by nitrogen-fixing cyanobacteria (Kollmen and Strieth, 2022). Moreover, cyanobacteria can optimize the mineralization of organic compounds and nutrient availability (Tarquinio et al., 2019). In this regard, the potential role of microbiota for holobiont health and restoration efficacy has been recently highlighted (Corinaldesi et al., 2023).

Our study also shows that the application of growth-promoters (particularly PGPB) on the fragments increases their survival and the formation of new roots and leaves. The use of PGPB on *C. nodosa* fragments could allow the re-employment in restoration interventions, also when they are found broken or stranded along the beaches, without damaging the natural populations. This could be particularly important for restoration purposes, as already observed for macroalgae (Marletta et al., 2024). In the Mediterranean Sea, the probability of detached fragments returning back to the sea is very low, also due to the limited tide excursion, and generally, these fragments dry up on the beach (Balestri et al., 2011), but can be salvaged and used directly as a source for restoration/mitigation efforts (Balestri et al. (2011); Marletta et al., 2024) to promote habitat restoration.

The implementation of these growth-promoters, particularly PGPB, represents a yet unexplored field for marine plant research and it can offer a new way to improve seagrass health, and resilience, and increase restoration success (Tarquinio et al., 2019), reducing the time and costs of plant maintenance in mesocosms and ensuring long-term transplant success (Pansini et al., 2024) speeding up the process of roots and leaves’ formation (Balestri and Bertini, 2003). Nature-based solutions relying on microbiome analyses (and also through omics approaches) enable health monitoring of transplanted organisms/metacommunities and potential identification/production of probiotics/bio-promoters to stabilize unhealthy conditions of transplants (Corinaldesi et al., 2023). The use of microbes in ecosystem restoration is gaining increasing attention (Farrell et al., 2020; Watson et al., 2022). Microbe-assisted restoration has been implemented for different purposes in both terrestrial and aquatic environments (Robinson et al., 2023): plants’ and animals’ health (Gao et al., 2010; Contos et al., 2021; Birnbaum and Trevathan-Tackett, 2023), nutrient cycling (Garcia and Kao-Kniffin, 2018; Singh Rawat et al., 2023), drought stress tolerance (Ma et al., 2020; Sarabi and Arjmand-Ghajur, 2021), hormone production in plants and animals (Eichmann et al., 2021), climate regulation (Wu et al., 2022), pollination (Martin et al., 2022) and phytoremediation in degraded habitats (Haldar and Ghosh, 2020; Sarker et al., 2023; Agrawal et al., 2024). This study could contribute to the knowledge of new protocols for the conservation and restoration of seagrass meadows to reverse their loss and to optimize both the biodiversity and ecosystem services they provide (Possingham et al., 2015). This is an important target for the “UN Decade on Ecosystem Restoration” (Waltham et al., 2020) and the EU Biodiversity Strategy for 2030, aiming at restoring ecosystems across land and sea, especially those with considerable value in terms of goods and services such as seagrasses (Costanza et al., 2014; Vassallo et al., 2013).

Recent scientific advancements indicate that marine habitat restoration is feasible and should be upscaled, but it is constrained by: 1) the high costs when compared with terrestrial restoration and 2) the potential impact on source populations. This is particularly critical for habitat-forming species, a key targets of marine ecosystem restoration. Due to their successful application on terrestrial plants, growth promoters can be useful to enhance the recovery also of marine plants. In particular, we suggest the use of growth promoters in stranded plants, which would otherwise be lost. This phenomenon is expected to become more frequent as a consequence of climate change, or due to the increasing occurrence of storms. The use of the plant promoters tested here could make stranded organisms an important resource of viable material to be used in transplanting interventions. The set up and implementation of restoration methodologies are particularly important in the framework of the Nature Restoration Regulation recently approved by the European Union, which has binding restoration targets also for marine habitats (at least 30% of the EU’s land and sea areas by 2030, 60% by 2040 and 90% by 2050).

## Data Availability

The original contributions presented in the study are included in the article/supplementary material. Further inquiries can be directed to the corresponding author.
